# Association of secondhand smoke exposure during pregnancy with left ventricle structure and function in offspring at 4 years old: a prospective birth cohort study

**DOI:** 10.1186/s12884-025-07636-7

**Published:** 2025-04-29

**Authors:** Bo Wang, Yongxuan Peng, Hualin Wang, Zhikang Xu, Bowen Du, Yiwei Niu, Zhuoyan Li, Zhi Wang, Qianchuo Wang, Caifang Xu, Shengju Yin, Yanan Lu, Jian Wang, Kun Sun

**Affiliations:** 1 Department of Pediatric Cardiology, Xinhua Hospital, Shanghai Jiao Tong University School of Medicine, Shanghai, 200092 China; 2 Institute for Cardiovascular Development and Regenerative Medicine, Xinhua Hospital, Shanghai Jiaotong University School of Medicine, Shanghai, China; 3 Ministry of Education-Shanghai Key Laboratory of Children’s Environmental Health, School of Public Health, Shanghai Jiao Tong University School of Medicine, Shanghai, 200025 China

**Keywords:** Secondhand smoke during pregnancy, Left ventricle structure, Prospective cohort study

## Abstract

**Background:**

The association of secondhand smoke exposure during pregnancy with childhood cardiac alterations remains insufficiently elucidated. This study aims to explore the correlation between maternal passive smoking during gestation with cardiac structure and function of offspring.

**Methods:**

1089 mother-offspring pairs from the Shanghai Birth Cohort were included. Information on secondhand smoke exposure during each trimester of pregnancy and baseline characteristics was documented during pregnancy via structured questionnaire. Subsequent follow-up assessments, encompassing anthropometric information and echocardiographic evaluation, were conducted from 2018 to 2021, when the children were 4 years old. Multiple linear regressions models were used to explore the association of secondhand smoke exposure during pregnancy with left ventricular measurements in early childhood.

**Results:**

Secondhand smoke exposure during pregnancy was correlated with increased left ventricle (LV) internal diameter in diastole [LVIDd; β = 0.38, 95%CI: (0.06, 0.70)] and in systole [LVIDs; β = 0.28, 95%CI: (0.02, 0.54)] adjusted for maternal and child characteristics. Specifically, maternal passive smoking in early pregnancy (≤ 12 weeks of gestation) showed a positive association with LV internal diameter in diastole [LVIDd; β = 0.46, 95%CI: (0.14, 0.79)], in systole [LVIDs; β = 0.35, 95%CI: (0.09, 0.60)], end diastolic volume [LVEDV; β = 1.45, 95%CI: (0.26, 2.63)] and end systolic volume [LVESV; β = 0.68, 95%CI: (0.18, 1.19)]. No significant association was observed between secondhand smoke exposure during pregnancy and LV function of offspring.

**Conclusion:**

Secondhand smoke exposure during pregnancy was correlated with subclinical alterations in LV dimensions of offspring, especially during the early stage of pregnancy. Further research is necessary to confirm our findings and to explore the long-term effect of these cardiac changes on later cardiovascular risks.

**Supplementary Information:**

The online version contains supplementary material available at 10.1186/s12884-025-07636-7.

## Introduction

Prenatal tobacco exposure has been identified as a potential precipitant of various detrimental effects on the offspring health. Existing literature underscores a correlation between maternal smoking and the development of fetus, encompassing compromised fetal growth [1, 2], perturbed brain development [3] and multiple birth congenital anomalies [4, 5]. Moreover, these deleterious effects may persist throughout the offspring’s childhood and give rise to a diverse spectrum of adverse consequences, including obesity [6], attention deficit hyperactivity disorder [7], fractures [8], asthma [9] and allergic responses, which aligns with the Developmental Origins of Health and Disease theory [10].

In the realm of cardiovascular health, prenatal tobacco exposure also manifests long-term detrimental effects on children. Current research in this field has primarily focused on its impact on vascular structure and function. Specifically, epidemiological studies have consistently demonstrated that maternal smoking during pregnancy is associated with elevated offspring systolic blood pressure [11–13], and increased artery intima-media thickness, an established subclinical marker of early atherosclerosis development [14–16]. However, evidence regarding the effects of prenatal tobacco exposure on cardiac structure and function remains limited. While a few studies have reported associations with reduced heart rate variability [17, 18], the relationship with left ventricular (LV) structure and function - direct indicators of future cardiovascular health- [19, 20] has not been systematically investigated.

While the correlation between maternal active smoking during pregnancy and the health of offspring is clearly clarified, the effect of maternal secondhand smoke (SHS) exposure on children is less widely known, especially on the cardiovascular health of offspring [18]. In contrast to active smoking, exposure to SHS is significantly more prevalent during pregnancy, which underscores the need for increased research and public health efforts to mitigate SHS risks in this vulnerable population [21, 22].

Therefore, we aimed to investigate the association of maternal passive smoking with offspring LV geometry and function at 4 years of age to explore the influence of SHS of pregnant women on early childhood cardiac structure and function and to identify the critical window of this impact. We hypothesized that maternal secondhand smoke exposure during pregnancy would be associated with subclinical alterations in offspring LV structure and function, and that early pregnancy, as a critical period for cardiac development, would represent a key window of susceptibility to these cardiovascular effects.

## Methods

### Participants

Our research is based on Shanghai Birth Cohort (SBC), which is an ongoing prospective cohort study conducted in six collaborating hospitals in Shanghai, China. Women at preconception or early pregnancy care clinics were recruited between 2013 and 2016 and a total of 3426 pregnancies were included. A detailed description of the cohort has been provided elsewhere [23]. Briefly, pregnant women were recruited from six participating hospitals in Shanghai. Inclusion criteria were: (1) maternal age ≥ 20 years; (2) at least one parent being a registered Shanghai resident; (3) intention to deliver at a participating hospital; and (4) willingness to provide written informed consent and participate in follow-up. After excluding miscarriages, stillbirths, and withdrawals, 2200 live-born children attended face-to-face follow-up at 4 years of age, while the remaining participants were followed up via telephone.

As part of the cohort, our study only included mother-offspring pairs with complete data on passive smoking during pregnancy. Multiple pregnancies, congenital anomalies, mothers with history of active smoking before pregnancy, and children who were uncooperative or lost to follow up were excluded (Figure S1). A total of 1089 mother-offspring pairs were included in this study. Study protocols were approved by the Institutional Review Board of Xinhua Hospital Affiliated to Shanghai Jiao Tong University School of Medicine (XHEC-C-2013-001-2). Written informed consent was obtained from all study participants.

### Maternal characteristics

The maternal self-reported questionnaires were conducted after enrollment and during pregnancy with the help of trained staff. Information on maternal demographic and sociodemographic characteristics (e.g. maternal income, race and education level) reproductive characteristics (e.g. parity, gestational week, delivery mode, birth weight and length), lifestyle factors (e.g. alcohol drinking status during pregnancy) were collected using structured questionnaires. The history of hypertensive disorders in pregnancy (HDP) and gestational diabetes mellitus (GDM) was collected using structured questionnaires or extraction of inpatient history from medical records. Pre-pregnancy weight and height, weight in the first trimester (≤ 12 weeks), and weight before delivery were measured at each clinical visit.

Information on passive smoking during pregnancy was collected based on self-reported questionnaire during first (≤ 16 week), second (24–28 weeks) and third trimesters of pregnancy (32–34 weeks). Passive smoking during a given trimester was defined as exposure to another person’s tobacco smoke for at least 15 min daily for more than one day per week either in home or public space [24, 25]. Overall exposure to secondhand smoke during pregnancy was categorized as “yes” if exposure occurred during any one of the three trimesters (early, mid, or late pregnancy). The details of questionnaires are provided in the supplementary material.

### Offspring characteristics

Anthropometric measurement was performed at the age of 4 in the offspring by a trained examiner. Height and weight were precisely measured to the nearest 0.1 cm and 0.1 kg respectively, with children removing shoes, standing upright, and dressed in lightweight clothing. BMI was calculated as body weight (kg) divided by height squared (m^2^).

Transthoracic echocardiography examinations were performed on the children by trained operators according to the American Society of Echocardiography recommendations [26] and using the Philip EPIQ7C (Philips Healthcare, Andover, USA) ultrasound that uses the X5-1 (1–5 MHz) or S8-3 (3–8 MHz) matrix-array transducers (Philips Healthcare, Andover, USA). Measurements of the left ventricle (LV) dimensions were acquired from two dimensional (2D)-guided M-mode echocardiograms, including the thickness of LV interventricular septum (IVSs and IVSd), posterior wall (LVPWs and LVPWd), and the internal diameter (LVIDs and LVIDd) of the LV during systole and diastole. LV ejection fraction (LVEF) and fractional shortening (FS) were calculated to evaluate the systolic function of LV. Relative wall thickness (RWT) was calculated by the sum of the thickness of the LV posterior wall in diastole (LVPWd) and interventricular septal thickness in diastole (IVSd), then divided by the internal LV diameter in diastole (LVIDd) [27]. LV mass (LVM) was calculated using the Devereux formula [28] and the LVM index (LVMI) was calculated using the formula: LVMI = LVM/Height^2.7^ [29].

### Statistical analysis

Data are presented as mean (SD), or as count (valid percentage, excluding missing values). Distributions of maternal and offspring characteristics were compared between mothers who were exposed to SHS and those who were not exposed to SHS during pregnancy. Two-tailed Student t test was used for continuous variables and Pearson χ2 test or Fisher exact test was used for categorical variables. *P* < 0.05 was considered statistically significant.

Multiple linear regression models were used to investigate the associations between maternal passive smoking status and offspring LV structure and function changes. Multiple comparisons were controlled using the Benjamini-Hochberg false discovery rate (FDR) procedure, with a significance threshold of FDR < 0.05. Covariates were chosen from the available data based on clinical expertise and existing research [30–32]. Moreover, a directed acyclic graph (DAG) was developed to further refine the selection of covariates (Figure S2) using the online tool (https://www.dagitty.net/). Three sets of models were constructed: the crude model was adjusted for none of the maternal factors or offspring factors. Model 1 was adjusted for maternal age at delivery, income, educational level, pre-pregnancy BMI, gender of children and postnatal SHS exposure. Model 2 was adjusted additionally for birthweight and children’s BMI at 4 years of age to explore their potential mediating effects.

To examine the robustness of the findings, we performed sensitivity analyses. First, we excluded mothers with GDM or HDP and then again investigated the associations between maternal passive smoking status and offspring LV structure and function changes. Second, we excluded children with obesity. The diagnosis of obesity was conducted according to the BMI reference for screening overweight and obesity in Chinese children [33]. All statistical analysis were carried out using the SPSS 27.0 software program (IBM Corp., Armonk, NY, USA) and R 4.3.1 (R Foundation for Statistical Computing).

## Results

### Subject characteristics

Demographic characteristics according to passive smoking status during pregnancy is shown in Table 1. Of the 1089 mother–child pairs, 533 (48.9%) mothers reported SHS exposure during pregnancy, with 458 (42.1%) reporting exposure during early pregnancy and 309 (28.4%) during mid/late pregnancy. Compared with women with no SHS exposure during pregnancy, women exposed to SHS had comparatively lower educational attainment and income level (*P* < 0.001), and had higher pre-pregnancy BMI (*P* = 0.018). Children of mothers exposed to SHS had higher gestational age, birthheight and weight at 4 years old. There were no significant differences in most baseline characteristics between the study population and the overall population (Table S1).

**Table 1:** Baseline characteristics of participants included in the study (*N* = 1089)

		Overall SHS exposure during pregnancy		
**Characteristic**	**Overall** (*n* = 1089)	No (*n* = 556)	Yes (*n* = 533)	**p-value**
**Mother characteristics**				
Maternal age, mean (SD), years	30.80 (3.45)	31.00 (3.36)	30.60 (3.53)	0.059
Han ethnic group, N (%)	1069 (98.2%)	542 (97.5%)	527 (98.9%)	0.200
Educational level, N (%)				**< 0.001**
≥Bachelor’s degree	793 (72.8%)	447 (80.4%)	346 (64.9%)	
< Bachelor’s degree Unreported	292 (26.8%) 4 (0.4%)	107 (19.2%) 2 (0.4%)	185 (34.7%) 2 (0.4%)	
Income, N (%),RMB/year				**< 0.001**
< 100,000	568 (52.2%)	289 (52.0%)	279 (52.3%)	
≥ 100,000	317 (29.1%)	184 (33.1%)	133 (25.0%)	
Unreported	204 (18.7%)	83 (14.9%)	121 (22.7%)	
BMI, mean(SD), kg/m^2^	21.60 (3.03)	21.39 (2.98)	21.82 (3.06)	**0.018**
HDP, N (%)	64 (5.9%)	23 (5.0%)	30 (6.9%)	0.200
GDM, N (%)	159 (14.6%)	79 (14.2%)	80 (15.0%)	0.708
Drink during pregnancy, N (%)	119 (10.9%)	51 (9.2%)	68 (12.7%)	0.058
**Children characteristics**				
Gender (Boy), N (%)	579 (53.2%)	293 (52.7%)	286 (53.7%)	0.800
Gestational age, mean (SD), weeks	39.17 (1.49)	39.06 (1.56)	39.30 (1.40)	**0.011**
Birth length, mean (SD), cm	49.89 (1.32)	49.81 (1.31)	49.99 (1.31)	**0.042**
Birthweight, mean (SD), g	3,354.9 (442.3)	3,332.2 (440.3)	3,379.3 (443.6)	0.088
Postnatal SHS exposure, N (%)				**< 0.001**
No	674 (61.9%)	404 (72.7%)	270 (50.7%)	
Yes	300 (27.6%)	102 (18.4%)	198 (37.1%)	
Unreported	115 (10.6%)	50 (9.0%)	65 (12.2%)	
Height at 4, mean (SD), cm	107.93 (4.88)	107.69 (4.69)	108.19 (5.06)	0.094
Weight at 4, mean (SD), kg	17.61 (2.77)	17.44 (2.67)	17.78 (2.85)	**0.040**
BMI at 4, mean (SD), kg/m^2^	15.06 (1.62)	14.98 (1.51)	15.14 (1.74)	0.100

The bold values were *P* < 0.05

For continuous variables, data are presented as mean SD and P value was calculated using student t test. For categorical variables, data are presented as number of participants (%) and differences between groups were calculated by Chi-square test

BMI Body mass index, HDP Hypertensive disorders in pregnancy, GDM Gestational diabetes mellitus

### Association of overall SHS exposure during pregnancy with cardiac parameters of children

Compared with children from mothers with no SHS exposure during pregnancy, children from mothers who were exposed to SHS during pregnancy had larger LVIDd, LVIDs and ESV (*P* = 0.024, *P* = 0.045, *P* = 0.048 respectively). Children of the passive smoking group had a trend of enlarged EDV, as the P value was close to 0.05. Correlation analysis of LV parameters (Figure S3) revealed strong associations among these structural measures (e.g., LVIDd-LVEDV: *r* = 0.98), supporting the consistency of these findings. The RWT and LV function were not significantly different between the two groups (Table 2).

**Table 2:** LV structure and function of children at 4 years old

		Overall SHS exposure during pregnancy		
	**Overall (** ***N*** ** = 1089)**	No (*n* = 556)	Yes (*n* = 533)	**p-value**
**LV structure**				
LVIDd (mm)	35.72 (2.56)	35.54 (2.57)	35.91 (2.55)	**0.024**
LVIDs (mm)	22.75 (1.99)	22.62 (1.98)	22.88 (2.00)	**0.045**
IVSd (mm)	3.82 (0.53)	3.81 (0.56)	3.83 (0.50)	0.673
IVSs (mm)	6.58 (0.99)	6.56 (1.01)	6.59 (0.97)	0.704
LVPWs (mm)	7.83 (0.97)	7.83 (1.00)	7.84 (0.94)	0.800
LVPWd (mm)	4.13 (0.59)	4.13 (0.64)	4.14 (0.54)	0.900
RWT (%)	22.39 (3.29)	22.48 (3.54)	22.29 (3.02)	0.356
EDV (ml)	53.82 (9.29)	53.25 (9.09)	54.40 (9.46)	0.051
ESV (ml)	17.87 (3.89)	17.63 (3.83)	18.12 (3.94)	**0.048**
LVMI (g/cm^2.7^)	26.17 (4.91)	26.07 (5.12)	26.27 (4.68)	0.517
**LV Function**				
SV (ml)	45.94 (12.87)	46.05 (12.91)	45.82 (12.85)	0.780
CO (L/min)	4.17 (1.20)	4.18 (1.24)	4.16 (1.17)	0.883
LVEF (%)	66.84 (4.14)	66.87 (4.09)	66.80 (4.18)	0.785
LVFS (%)	36.31 (3.25)	36.33 (3.25)	36.28 (3.26)	0.814
E/A	1.82 (0.36)	1.81 (0.35)	1.82 (0.37)	0.771
Tei index (%)	43.70 (6.64)	43.39 (6.44)	44.01 (6.83)	0.132

The bold values were *P* < 0.05

For continuous variables, data are presented as mean SD and P value was calculated using student t test

LV Left ventricle, LVIDd LV internal diameter in diastole, LVIDs LV internal diameter in systole, IVSs Interventricular septal thickness in systole, IVSd Interventricular septal thickness in diastole, LVPWs LV posterior wall thickness in systole, LVPWd LV posterior wall thickness in diastole, EDV End diastolic volume, ESV End systolic volume, IVSd Interventricular septal thickness in diastole, IVSs Interventricular septal thickness in systole, RWT Relative wall thickness, LVMI LV mass index, SV Stroke volume, CO Cardiac output, LVEF LV Ejection fraction, LVFS LV fractional shortening

To explore the association between SHS exposure during pregnancy and cardiac parameters of children, we performed linear regression analysis (Table 3). Overall exposure to SHS during pregnancy (No or Yes) was found to be correlated with larger LV diameters [LVIDd: β = 0.38, 95% CI: (0.06, 0.70), LVIDs: β = 0.28, 95% CI: (0.02, 0.54)] and volume [EDV: β = 1.18, 95% CI: (0.01,2.35), ESV: β = 0.55, 95% CI: (0.05, 1.05)] after adjusting for maternal and children’s characteristics. And the association become attenuated but still significant after being additionally adjusted for birthweight and BMI at 4 years old [LVIDd: β = 0.35, 95% CI: (0.04, 0.66), LVIDs: β = 0.24, 95% CI: (-0.01, 0.49)].

**Table 3:** Associations of maternal smoking during pregnancy with cardiac structures and function in 4-year-old children

	Overall SHS exposure during pregnancy^a^		
	Crude Model	Model 1	Model 2
**LV structure**			
LVIDd (mm)	**0.37 (0.05**,** 0.69)**	**0.38 (0.06**,** 0.70)**	**0.35 (0.04**,** 0.66)**
LVIDs (mm)	**0.25 (0.01**,** 0.50)**	**0.28 (0.02**,** 0.54)**	0.24 (-0.01, 0.49)
IVSd (mm)	0.01 (-0.05, 0.08)	-0.01 (-0.08, 0.06)	0.01 (-0.05, 0.08)
IVSs (mm)	0.02 (-0.10, 0.15)	0.00 (-0.13, 0.12)	0.02 (-0.11, 0.14)
LVPWd (mm)	0.01 (-0.11, 0.13)	-0.02 (-0.15, 0.10)	-0.01 (-0.14, 0.11)
LVPWs (mm)	0.00 (-0.07, 0.08)	-0.01 (-0.09, 0.06)	0.00 (-0.07, 0.08)
RWT (%)	-0.19 (-0.60, 0.22)	-0.33 (-0.76, 0.10)	-0.18 (-0.59, 0.23)
EDV (ml)	1.15 (-0.01, 2.31)	**1.18 (0.01**,** 2.35)**	1.06 (-0.08, 2.20)
ESV (ml)	**0.49 (0.01**,** 0.97)**	**0.55 (0.05**,** 1.05)**	0.47 (-0.02, 0.96)
LVMI (g/cm^2.7^)	0.20 (-0.41, 0.81)	0.14 (-0.50, 0.77)	0.32 (-0.29, 0.94)
**LV Function**			
SV (ml)	-0.23 (-1.86, 1.39)	-0.12 (-1.79, 1.54)	0.03 (-1.64, 1.69)
CO (L/min)	-0.01 (-0.16, 0.14)	0.00 (-0.16, 0.15)	0.01 (-0.15, 0.17)
LVEF (%)	-0.07 (-0.59, 0.44)	-0.14 (-0.68, 0.41)	-0.10 (-0.65, 0.46)
LVFS (%)	-0.05 (-0.45, 0.36)	-0.09 (-0.52, 0.33)	-0.07 (-0.50, 0.37)
E/A	0.01 (-0.04, 0.05)	0.00 (-0.04, 0.05)	-0.01 (-0.05, 0.04)
Tei index (%)	0.62 (-0.19, 1.43)	0.40 (-0.45, 1.25)	0.54 (-0.34, 1.42)

The beta values and 95% CI derived from the multiple linear regression models are presented in the table. Bold: *P* < 0.05; #: FDR < 0.05

Model 1: Adjusted for maternal age, income, educational level, pre-pregnancy body mass index, gender of children and postnatal SHS exposure

Model 2: Model 2 additionaly adjusted for birthweight and BMI at 4 years old

LV Left ventricle, LVIDd LV internal diameter in diastole, LVIDs LV internal diameter in systole, IVSs Interventricular septal thickness in systole, IVSd Interventricular septal thickness in diastole, LVPWs LV posterior wall thickness in systole, LVPWd LV posterior wall thickness in diastole, EDV End diastolic volume, ESV End systolic volume, IVSd Interventricular septal thickness in diastole, IVSs Interventricular septal thickness in systole, RWT Relative wall thickness, LVMI LV mass index, SV Stroke volume, CO Cardiac output, LVEF LV Ejection fraction, LVFS LV fractional shortening

^a^Reference category are mothers with no SHS exposure during pregnancy

### SHS exposure at different stages of pregnancy and LV structure and function of offspring

Given the critical role of the early gestational period in fetal cardiac development, we divided the pregnancy duration into early and mid/late stages to investigate their respective impacts on the cardiac structure and function of the offspring. Positive association was found between SHS exposure during early stage of pregnancy and larger LV diameters [LVIDd: β = 0.46, 95% CI: (0.14, 0.79), LVIDs: β = 0.35, 95% CI: (0.09, 0.60)] and volume [EDV: β = 1.45, 95% CI: (0.26,2.63), ESV: β = 0.68, 95% CI: (0.18, 1.19)] after adjusting for maternal and children’s characteristics. Similarly, the association was attenuated but still significant after additionally adjusting for BMI at 4 years of age (Table 4). However, SHS exposure during mid/late pregnancy was not significantly correlated with LV geometric parameters in children. To further disentangle the independent effects of early and mid/late pregnancy SHS exposure, we categorized exposure into four mutually exclusive groups: no exposure, early pregnancy only, mid/late pregnancy only, and exposure throughout pregnancy. Results showed that early pregnancy exposure alone (without mid/late exposure) was significantly associated with increased LVIDd, LVIDs, LVEDV, and LVESV, confirming the robustness of our findings (Table S2). After adjustment for multiple comparisons, the associations between passive smoking during early pregnancy and LVIDd, LVIDs, and ESV remained significant.

**Table 4:** Associations of maternal smoking during different stages of pregnancy with cardiac structures and function in 4-year-old children

	Maternal passive smoking during early pregnancy^a^			Maternal passive smoking during mid/late pregnancy^a^		
	Crude Model	Model 1	Model 2	Crude Model	Model 1	Model 2
**LV structure**						
LVIDd (mm)	**0.40 (0.08**,** 0.73)**	**0.46 (0.14**,** 0.79)**^**#**^	**0.42 (0.10**,** 0.73)**	-0.30 (-0.66, 0.05)	-0.30 (-0.65, 0.05)	-0.26 (-0.60, 0.09)
LVIDs (mm)	**0.29 (0.04**,** 0.54)**	**0.35 (0.09**,** 0.60)**^**#**^	**0.26 (0.01**,** 0.52)**	-0.17 (-0.45, 0.10)	-0.15 (-0.43, 0.13)	-0.12 (-0.40, 0.15)
IVSd (mm)	0.01 (-0.06, 0.07)	-0.01 (-0.08, 0.06)	0.00 (-0.06, 0.07)	0.02 (-0.06, 0.09)	-0.01 (-0.08, 0.07)	0.02 (-0.05, 0.10)
IVSs (mm)	0.03 (-0.09, 0.16)	0.01 (-0.11, 0.14)	0.02 (-0.11, 0.15)	-0.04 (-0.17, 0.10)	-0.08 (-0.21, 0.06)	-0.05 (-0.19, 0.09)
LVPWd (mm)	0.03 (-0.09, 0.15)	0.01 (-0.12, 0.13)	0.01 (-0.11, 0.14)	-0.02 (-0.16, 0.11)	-0.07 (-0.21, 0.07)	-0.02 (-0.16, 0.12)
LVPWs (mm)	-0.01 (-0.09, 0.06)	-0.03 (-0.10, 0.05)	-0.02 (-0.10, 0.06)	0.01 (-0.07, 0.09)	-0.01 (-0.10, 0.07)	0.02 (-0.06, 0.10)
RWT (%)	-0.28 (-0.70, 0.13)	**-0.43 (-0.87**,** 0.00)**	-0.33 (-0.74, 0.09)	0.25 (-0.21, 0.70)	0.10 (-0.37, 0.57)	0.28 (-0.17, 0.73)
EDV (ml)	**1.27 (0.10**,** 2.44)**	**1.45 (0.26**,** 2.63)**	**1.27 (0.11**,** 2.42)**	-1.14 (-2.42, 0.14)	-1.15 (-2.43, 0.12)	-0.99 (-2.25, 0.27)
ESV (ml)	0.57 (0.08, 1.06)	**0.68 (0.18**,** 1.19)**^**#**^	**0.52 (0.02**,** 1.02)**	-0.39 (-0.93, 0.14)	-0.34 (-0.89, 0.20)	-0.28 (-0.82, 0.26)
LVMI (g/cm^2.7^)	0.27 (-0.34, 0.89)	0.27 (-0.37, 0.91)	0.37 (-0.25, 1.00)	-0.17 (-0.85, 0.51)	-0.27 (-0.96, 0.43)	0.00 (-0.68, 0.69)
**LV Function**						
SV (ml)	-0.67 (-2.31, 0.97)	-0.56 (-2.24, 1.13)	-0.61 (-2.30, 1.07)	-0.73 (-2.52, 1.07)	-0.44 (-2.25, 1.37)	0.43 (-1.40, 2.26)
CO (L/min)	-0.08 (-0.23, 0.08)	-0.07 (-0.23, 0.08)	-0.08 (-0.24, 0.08)	0.01 (-0.16, 0.17)	0.04 (-0.12, 0.21)	0.11 (-0.06, 0.29)
LVEF (%)	-0.10 (-0.62, 0.43)	-0.17 (-0.72, 0.38)	-0.04 (-0.60, 0.53)	-0.03 (-0.60, 0.54)	-0.09 (-0.68, 0.51)	-0.16 (-0.77, 0.45)
LVFS (%)	-0.06 (-0.47, 0.35)	-0.11 (-0.54, 0.32)	0.00 (-0.44, 0.44)	-0.09 (-0.54, 0.36)	-0.12 (-0.59, 0.34)	-0.19 (-0.67, 0.29)
E/A	0.03 (-0.02, 0.07)	0.03 (-0.02, 0.07)	0.02 (-0.03, 0.06)	-0.01 (-0.06, 0.04)	-0.02 (-0.07, 0.03)	-0.03 (-0.08, 0.02)
Tei index (%)	0.65 (-0.17, 1.47)	0.40 (-0.47, 1.26)	0.55 (-0.34, 1.45)	-0.08 (-0.98, 0.82)	-0.23 (-1.16, 0.70)	-0.10 (-1.07, 0.88)

The beta values and 95% CI derived from the multiple linear regression models are presented in the table. Bold: *P* < 0.05; #: FDR < 0.05

Model 1: Adjusted for maternal age, income, educational level, pre-pregnancy body mass index, gender of children and postnatal SHS exposure

Model 2: Model 2 additionaly adjusted for birthweight and BMI at 4 years old

LV Left ventricle, LVIDd LV internal diameter in diastole, LVIDs LV internal diameter in systole, IVSs Interventricular septal thickness in systole, IVSd Interventricular septal thickness in diastole, LVPWs LV posterior wall thickness in systole, LVPWd LV posterior wall thickness in diastole, EDV End diastolic volume, ESV End systolic volume, IVSd Interventricular septal thickness in diastole, IVSs Interventricular septal thickness in systole, RWT Relative wall thickness, LVMI LV mass index, SV Stroke volume, CO Cardiac output, LVEF LV Ejection fraction, LVFS LV fractional shortening

^a^Reference category are mothers with no SHS exposure at each period

Similar associations were found between SHS during different stages of pregnancy and LV structure and function of offspring after excluding mothers with HDP or GDM (*N* = 196), and replacing BMI with BSA in model 2 (Table S3, S4).

## Discussion

In this prospective study, it was determined that prenatal exposure to secondhand smoke was associated with LV morphologic changes in 4-year-old offspring. The early stage of pregnancy was the critical window during which the association is most evident.

Tobacco use represents a worldwide concern. As a predominant tobacco-consuming nation, the issue of smoking in China has persistently garnered considerable scrutiny. The latest WHO report on the global tobacco epidemic showed that the age-standardized daily smoking prevalence of people aged 15 years or older was 21% in China in 2021 [34]. Such high prevalence of tobacco use exacerbates the risk of SHS exposure among children and adolescents. Any level of exposure to tobacco smoke is considered unsafe, posing health risks at all life stages, even in prenatal period. Prenatal tobacco exposure is a quintessential example of adverse early-life exposures in the DOHaD theory, offering no benefits but only detriments [10, 35]. Despite a large body of literatures on maternal active smoking [18], studies on SHS exposure during pregnancy and children’s cardiovascular health are limited, which is a more common phenomenon [21, 36]. Given that SHS involves exposure to a spectrum of tobacco toxins comparable to those encountered by active smokers, although at reduced concentrations, it is probably that SHS exposure during pregnancy leads to similar adverse outcomes but with lower levels of risk. Therefore, our study concentrated on the correlation between SHS exposure of non-smoking pregnant women and the cardiac structure and function of offspring, rooted in a large prospective birth cohort. Significant association was found between overall maternal passive smoking during pregnancy and LV diameters and volumes of offspring. Our study also discerned that early pregnancy was the critical window during which maternal passive smoking exerts the most pronounced impact on the fetal heart.

In this study, we found that the overall SHS exposure rate during pregnancy amounted to 48.9% based on maternal self-reports, which is consistent with prior studies in developed areas of China [37]. This alarmingly high proportion underscores the need of both smoking reduction and education of family members and health care providers, even in developed areas. Pregnant women exposed to SHS exhibited lower educational attainment and income levels, indicating a demographic that deserves enhanced health education efforts.

Maternal smoking during pregnancy is an important factor influencing cardiac development. Research has demonstrated that both active and passive maternal smoking are associated with an elevated incidence of congenital heart defects [4, 38]. However, few studies have documented the effect of SHS exposure in pregnancy on child cardiac structure and function beyond infancy. The only available study examining parental smoking during pregnancy and offspring cardiac structure reported an isolated association between paternal smoking and increased aortic root diameter in 6-year-olds, while finding no significant relationships with left ventricular mass (LVM) or fractional shortening (LVFS) - a pattern consistent with our observations [12]. However, parameters pertaining to other structures and functions of the left ventricle have not been explored. In our investigation, a thorough echocardiographic examination was carried out to comprehensively assess cardiac structure and function in children. The positive correlation between maternal passive smoking in pregnancy and LV size, is first discovered in our study.

LV diameters (LVIDd & LVIDs) and volumes (EDV & ESV) are crucial in evaluating ventricular patterns, encompassing ventricular dilation and hypertrophic responses, frequently observed in pathologies such as dilated cardiomyopathy [39] or hypertensive heart disease [40]. Our study suggests that SHS exposure in utero is correlated with enlarged LV of children, characterized by increased LV diameters and volumes, even after correction for maternal and children’s characteristics or exclusion of children with obesity. Enlarged LV size is a marker of adverse cardiac remodeling. In pediatric populations, increased LV size is associated with a poor prognosis in cardiomyopathies [41, 42]. In adult populations, LV diameter may contribute to risk stratification for sudden cardiac death independent of LV ejection fraction [41, 43]. Previous studies have established the association between SHS and cardiovascular health of children, including blood pressure [44–46], cardiac remodeling [47], and risks for multiple cardiovascular diseases [48, 49]. However, these studies were mostly cross-sectional studies focusing on the adverse effects of SHS after birth, while our findings provided initial data at the early stage of life. Our study advances the understanding of the adverse effects of SHS to the intrauterine stage, which was previously under underestimated.

The critical window has also been identified during which the association between prenatal SHS exposure and fetal cardiac development is most evident. The results demonstrated a significant association between SHS exposure during early pregnancy and cardiac remodeling in offspring, which may align with two critical biological phenomena. First, this period (3rd-8th week) represents the vital phase of cardiac morphogenesis when fundamental heart structures form and undergoes looping to establish the basic four-chamber structure. Second, during this early developmental phase, the placental barrier remains structurally and functionally immature, with incomplete formation of the trophoblast layer and underdeveloped tight junctions between cells [50, 51]. In contrast, exposure in mid/late pregnancy appears to have a lesser impact on cardiac structural development. By this stage, the heart has largely completed its morphogenesis, and the mature placental barrier reduces direct fetal toxin exposure. Additionally, fetal adaptive mechanisms such as heart rate modulation [52] and placental compensation [53] may mitigate the adverse effects of SHS exposure in later pregnancy. These results are consistent with prior research linking parental smoking to congenital heart defects [4, 38], while additionally indicating that first-trimester smoke exposure may affect both initial cardiac development and later remodeling pathways. Our findings emphatically advocate for extra surveillance for cardiac remodeling in the children, when their mothers were at high exposure of smoking.

The biological mechanisms linking prenatal smoking exposure to alterations in childhood cardiac structure and function remain somewhat obscure. A range of smoking-related substances could be implicated in this association. Nicotine is a key addictive component of all tobacco products and a significant teratogen which leads to reduced placental blood flow and lower oxygen levels in the fetus [54]. In mouse models, prenatal nicotine exposure were found to induce cardiovascular malformation and LV hypertrophy of offspring [55, 56]. The potential mechanisms might include higher levels of elevate reactive oxygen species (ROS) and epigenetic alteration [57]. Previous studies have suggested that maternal smoking may elevate ROS in offspring [58], with prenatal nicotine exposure potentially maintaining these effects long-term [59, 60]. These oxidative stress responses might interfere with crucial cardiac developmental pathways [61], possibly affecting progenitor cell differentiation and myocardial energy metabolism [62]. Moreover, emerging evidence suggests ROS may participate in epigenetic regulation of cardiac-related genes, potentially influencing postnatal cardiac development [62, 63]. Additional smoking-related toxins, like carbon monoxide and cadmium, could also contribute to decreased placental and fetal perfusion, as well as cardiovascular responses [64]. Further investigations are warranted to identify precise effects of various toxins contained in tobacco on cardiac structure in later life.

### Strengths and limitations

Our study has several strengths. First of all, it’s a prospective cohort study with a relatively large number of participants, enhancing the confidence of results. Additionally, with comprehensive cardiac assessment data in childhood, our research provides the initial evidence about the association between SHS exposure during early stage of life and cardiac parameters in early childhood, hinting that the 1st trimester of pregnancy is a crucial period. Lastly, standardized methodology, comprehensive anthropometric data and a variety of confounding variables are also among the strengths.

There are also some limitations in this study. First of all, the information about maternal passive smoking were collected by self-reports, which is not as precise as quantitative data. Moreover, maternal cardiovascular disease history could not be adjusted for in the analyses due to unavailability of these data. Lastly, there may exist a selection bias in our research. Our study was conducted using data from SBC, where most of the families were from urban areas and typically possessed relatively high education and socioeconomic status [23]. Our findings necessitate additional verification in other populations to reinforce their validity.

## Conclusion

Our findings indicate an association between prenatal secondhand smoke (SHS) exposure and increased left ventricular (LV) diameters and volumes in 4-year-old children. These results highlight the potential influence of SHS on early cardiac development. Further research is essential to confirm these findings and to explore the long-term cardiovascular implications of such alterations.

## Electronic supplementary material

Below is the link to the electronic supplementary material.

Supplementary Material 1

## Data Availability

Deidentified individual participant data will be made available, in addition to study protocols, the statistical analysis plan, and the informed consent form. The data will be made available upon publication to researchers who provide a methodologically sound proposal for use in achieving the goals of the approved proposal. Proposals should be submitted to wangjian@xinhuamed.com.cn.
