# Supplementary material for: Association of secondhand smoke exposure during pregnancy with left ventricle structure and function in offspring at 4 years old: a prospective birth cohort study
Source: BMC Pregnancy Childbirth. 2025 Apr 29;25:515. doi: 10.1186/s12884-025-07636-7 (PMC12039129; doi:10.1186/s12884-025-07636-7)
Supplement: Supplementary file 1 — Supplementary Material 1 [file 12884_2025_7636_MOESM1_ESM.docx]

**Supplementary Materials**

**Association of Secondhand Smoke exposure During Pregnancy with Left Ventricle Structure and Function in Offspring at 4 Years Old：A Prospective Birth Cohort Study**

Bo Wang, Yongxuan Peng, Hualin Wang, Zhikang Xu, Bowen Du, Yiwei Niu, Zhuoyan Li, Zhi Wang, Qianchuo Wang, Caifang Xu, Shengju Yin, Yanan Lu, Jian Wang, Kun Sun

CONTENTS

**Variable Definition in Questionnaire**

**Supplemental Figure 1.** Flowchart of the selection of study participants from the Shanghai Birth Cohort

**Supplemental Figure 2.** Directed Acyclic Graph (DAG) of the SHS exposure during pregnancy with LV structure and function of children.

**Supplemental Figure 3.** The Pearson correlations between LV measures.

**Supplemental Table 1.** Sensitivity analysis: Exclude mothers with GDM or HDP (N = 893)

**Supplemental Table 2.** Sensitivity analysis: Replacing BMI with Body Surface Area (BSA) in Model 2 (N = 1089)

**Supplemental Table 3.** Association Between Prenatal SHS Exposure and Offspring Cardiac Structure by Mutually Exclusive Exposure Timing Categories

**Variable Definition in Questionnaire**

**Exposure**

Passive smoking status during pregnancy

Questionnaires were administered to pregnant women during early (≤16week), mid (24-28 weeks), and late pregnancy (32-34 weeks) to assess smoking exposure. Questions used in evaluating the history of passive smoking are listed as follows.

1.1 Does your husband smoke? 0=No;1=Yes;99=Refuse to answer

1.2 If yes, how many cigarettes does he smoke indoors per day at home? 0 = 0 cigarettes; 1 = 1-4 cigarettes; 2 = >5 cigarettes; 3 = ≥10 cigarettes; 99 = Don't know

2.1 Does any other family member living with you smoke? 0=No; 1=Yes; 99=Refuse to answer

2.2 If yes, how many cigarettes does he or she smoke indoors per day at home? 0 = 0 cigarettes; 1 = 1-4 cigarettes; 2 = >5 cigarettes; 3 = ≥10 cigarettes; 99 = Don't know

3.1 Do colleagues in the same office smoke at work? 0=No; 1=Yes; 99=Refuse to answer

3.2 If yes, how many cigarettes does he or she smoke indoors per day at home? 0 = 0 cigarettes; 1 = 1-4 cigarettes; 2 = >5 cigarettes; 3 = ≥10 cigarettes; 99 = Don't know

4. How long are you exposed to secondhand smoke each day?

Passive smoking during a given trimester was defined as a "yes" response to any of questions 1.1, 2.1, or 3.1, combined with a reported daily exposure duration of >15 minutes in question 4. Otherwise, exposure was classified as negative or unknown. A woman was considered exposed to passive smoking during pregnancy if she met the criteria for at least one trimester.

**Covariate**

Household Income

The household income level was assessed using the following questions. Based on the distribution of responses, participants were dichotomized into two groups using a cutoff of ≥100,000 yuan. This binary income variable was included in the regression models.

What was your household income last year? 1= < 10000/year ; 2= 10000-3000/year; 3=30000-50000/year; 4=50000-100000/year;5=100000-150000/year ;6=150000-300000/year ;7=300000-1000000/year; 8= ≥1000000/year; 99=refuse to answer/unknown.

Maternal educational level

Education level was assessed using the following questions. Participants were categorized into two groups based on attainment of a bachelor’s degree, and this binary variable was included in the regression models.

What is your highest degree? 0=Didn't go to school; 1=primary school; 2=junior high school; 3=senior high school/technical school; 4=college; 5=Bachelors’ degree; 6=Postgraduate and above; 99 refuse to answer.

Drinking during pregnancy

Alcohol drinking history was assessed using the following questions. Participants were classified as positive if they reported “occasional” or “regular” alcohol consumption during any trimester; otherwise, they were classified as negative or unknown. This categorical variable was included in the regression models.

1.In the past 3 months, do you often drink alcohol? 0=Never; 1=Occasional (special occasion/holiday); 2=Regularly; 98=Refuse to answer (Asked in the first trimester)

2.Have you drunk alcohol since the last questionnaire interview? 0=Never; 1=Occasional (special conditions/holiday); 2=Regularly; 98=Refuse to answer (Asked in the second and third trimesters)

Postnatal Passive Smoking Exposure

Questionnaires were administered to parents when the children were 2 years old to assess their exposure to postnatal passive smoking. Participants were classified as positive if they reported “occasionally” or “frequently”; otherwise, they were classified as negative or unknown. This categorical variable was included in the regression models.

Has the child been exposed to secondhand smoke since birth?
1 = No, 2 = Yes, occasionally, 3 = Yes, frequently.


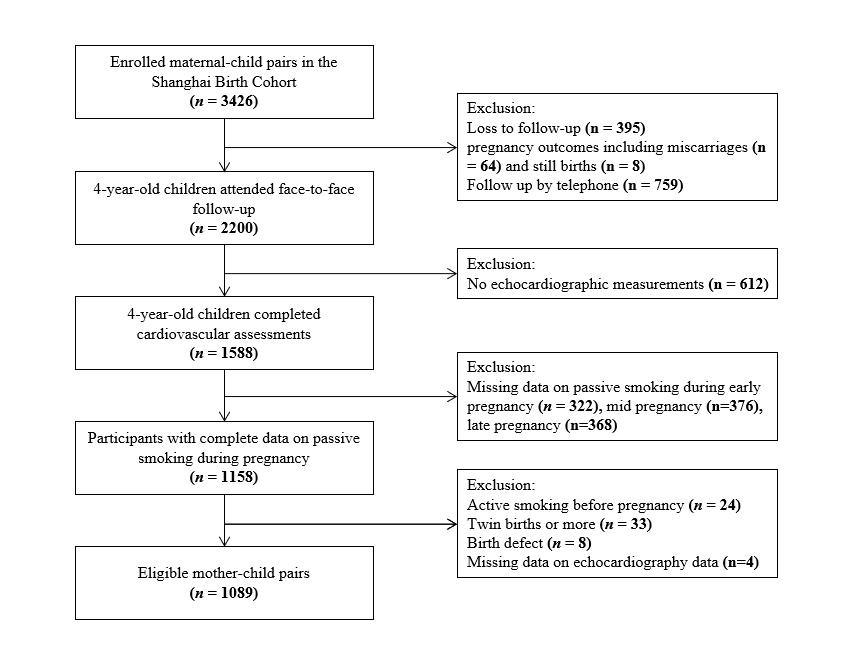


Figure S1 Flowchart of the selection of study participants from the Shanghai Birth Cohort


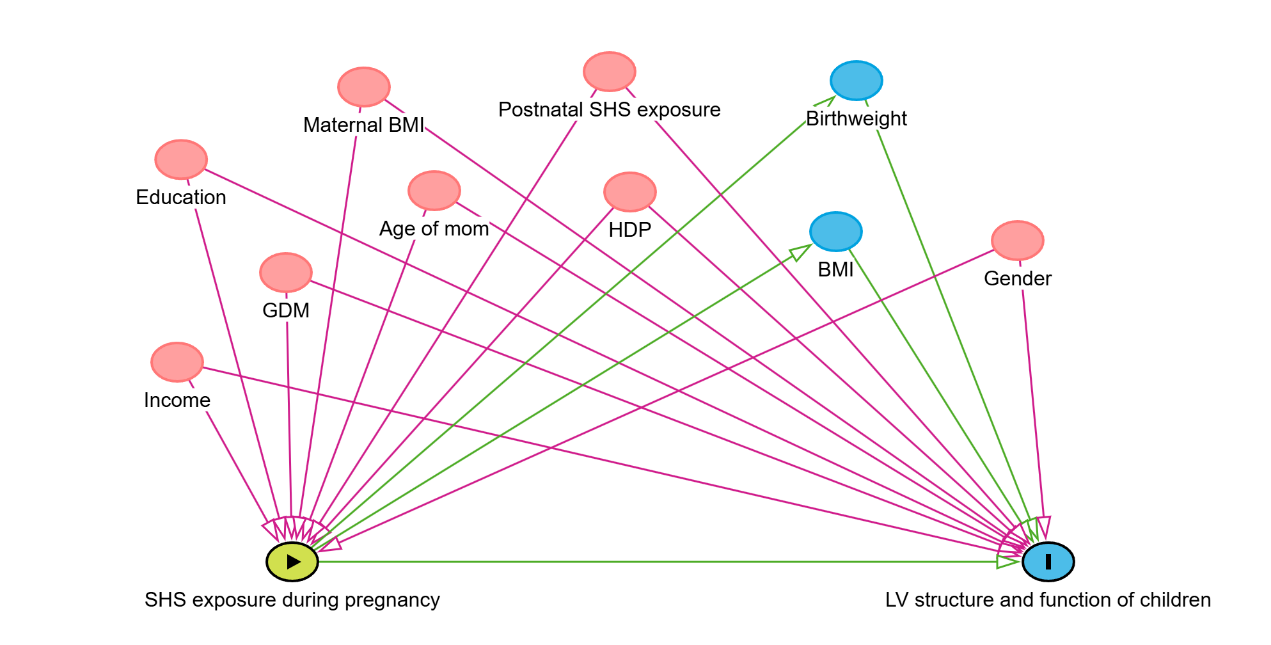


Figure S2. Directed Acyclic Graph (DAG) of the SHS exposure during pregnancy with LV structure and function of children.

BMI: body mass index, HDP: hypertensive disorders in pregnancy, GDM: gestational diabetes mellitus.


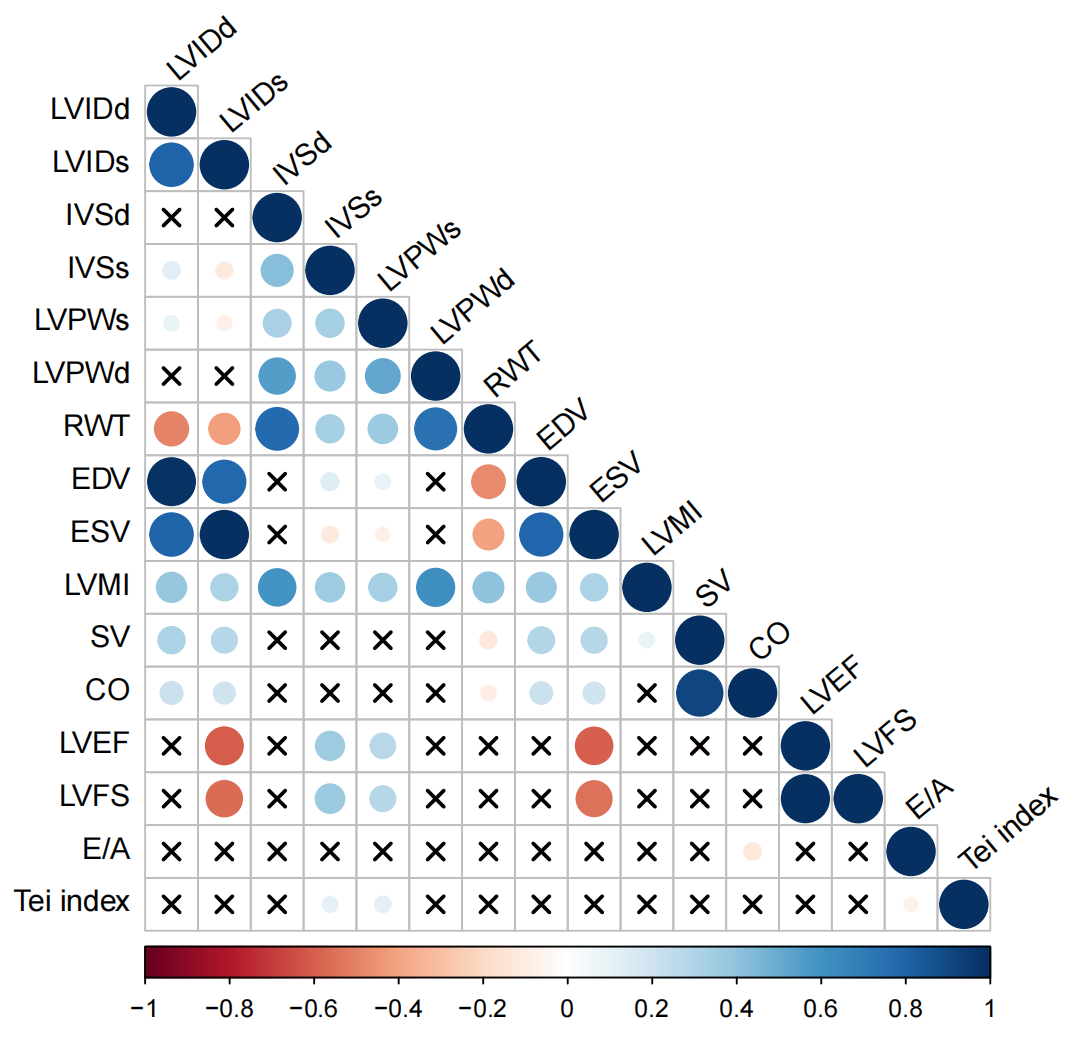


Figure S3. The Pearson correlations between LV measures. The size and color of circles represents the Pearson coefficients.

×: P > 0.05

LV Left ventricle, LVIDd LV internal diameter in diastole, LVIDs LV internal diameter in systole, IVSs Interventricular septal thickness in systole, IVSd Interventricular septal thickness in diastole, LVPWs LV posterior wall thickness in systole, LVPWd LV posterior wall thickness in diastole, EDV End diastolic volume, ESV End systolic volume, IVSd Interventricular septal thickness in diastole, IVSs Interventricular septal thickness in systole, RWT Relative wall thickness, LVMI LV mass index, SV Stroke volume, CO Cardiac output, LVEF LV Ejection fraction, LVFS LV fractional shortening.

Table S1 Comparison of Baseline Characteristics Between the Study Participants and the Overall Population

| **Characteristic** | **Participants** | **Initial population** | **p-value** |
| --- | --- | --- | --- |
| **Mother characteristics** | n =1089 | n = 3426 |  |
| Maternal age, mean (SD), years | 30.80 (3.45) | 28.72 (3.81) | < 0.001 |
| Han ethnic group, N (%) | 1069 (98.2%) | 3368 (98.3%) | 0.855 |
| Educational level, N (%) |  |  | < 0.001 |
| ≥Bachelor’s degree | 793 (72.8%) | 2182 (63.7%) |  |
| < Bachelor’s degree | 292 (26.8%) | 1227 (35.8%) |  |
| Unreported | 4 (0.4%) | 17 (0.5 %) |  |
| Income, N (%), RMB/year |  |  | 0.911 |
| <100000 | 568 (52.2%) | 1775 (51.8%) |  |
| ≥100000 | 317 (29.1%) | 1020 (29.8%) |  |
| Unreported | 204 (18.7%) | 631 (18.4%) |  |
| BMI, mean (SD), kg/m^2^ | 21.60 (3.03) | 21.52 (3.30) | 0.264 |
| HDP, N (%) | 64 (5.9%) | 207 (6.0%) | 0.812 |
| GDM, N (%) | 159 (14.6%) | 484 (14.1%) | 0.565 |
| **Children characteristics** | n = 1089 | n = 1588 |  |
| Gender (Boy), N (%) | 579 (53.2%) | 832 (52.7%) | 0.735 |
| Gestational age, mean (SD), weeks | 39.17 (1.49) | 39.04 (1.62) | 0.158 |
| Birth length, mean (SD), cm | 49.89 (1.32) | 49.83 (1.39) | 0.441 |
| Birthweight, mean (SD), g | 3,354.9 (442.3) | 3347.7 (447.1) | 0.962 |
| Height at 4, mean (SD), cm | 107.93 (4.88) | 108.12 (4.79) | 0.297 |
| Weight at 4, mean (SD), kg | 17.61 (2.77) | 17.80 (2.86) | 0.086 |
| BMI at 4, mean (SD), kg/m^2^ | 15.06 (1.62) | 15.16 (1.68) | 0.122 |

For continuous variables, data are presented as mean SD and P value was calculated using student t test. For categorical variables, data are presented as number of participants (%) and differences between groups were calculated by Chi-square test.

BMI Body mass index

Table S2. Sensitivity analysis: Association Between Prenatal SHS Exposure and Offspring Cardiac Structure by Mutually Exclusive Exposure Timing Categories

|  | Passive smoking during early pregnancy only^a^ (N=224) | | | Passive smoking during mid/late pregnancy only^a^ (N=75) | | | Passive smoking throughout pregnancy^a^ (N=234) | | |
| --- | --- | --- | --- | --- | --- | --- | --- | --- | --- |
|  | crude model | model 1 | model 2 | crude model | model 1 | model 2 | crude model | model 1 | model 2 |
| **LV structure** |  |  |  |  |  |  |  |  |  |
| LVIDd (mm) | **0.91 (0.50, 1.32) ^#^** | **0.90 (0.49, 1.31) ^#^** | **0.81 (0.41, 1.20) ^#^** | 0.09 (-0.55, 0.72) | -0.03 (-0.64, 0.58) | -0.01 (-0.61, 0.58) | **0.89 (0.14, 1.64)** | **0.83 (0.09, 1.57)** | 0.60 (-0.13, 1.32) |
| LVIDs (mm) | **0.59 (0.27, 0.91) ^#^** | **0.60 (0.27, 0.92) ^#^** | **0.50 (0.18, 0.82) ^#^** | 0.02 (-0.48, 0.51) | -0.05 (-0.53, 0.44) | 0.05 (-0.43, 0.53) | **0.74 (0.15, 1.32)** | **0.74 (0.15, 1.33)** | 0.47 (-0.11, 1.05) |
| IVSd (mm) | 0.01 (-0.08, 0.09) | -0.01 (-0.10, 0.08) | 0.00 (-0.08, 0.09) | 0.03 (-0.10, 0.16) | 0.01 (-0.12, 0.14) | 0.04 (-0.08, 0.17) | 0.04 (-0.12, 0.20) | -0.01 (-0.17, 0.15) | 0.02 (-0.13, 0.18) |
| IVSs (mm) | 0.08 (-0.08, 0.24) | 0.07 (-0.09, 0.23) | 0.07 (-0.09, 0.23) | -0.02 (-0.27, 0.23) | -0.05 (-0.29, 0.19) | 0.00 (-0.24, 0.24) | 0.03 (-0.27, 0.32) | 0.00 (-0.30, 0.29) | 0.05 (-0.25, 0.34) |
| LVPWd (mm) | 0.04 (-0.11, 0.20) | 0.03 (-0.13, 0.19) | 0.00 (-0.16, 0.15) | -0.06 (-0.30, 0.18) | -0.11 (-0.34, 0.13) | -0.09 (-0.33, 0.14) | -0.16 (-0.45, 0.13) | -0.27 (-0.55, 0.02) | -0.23 (-0.52, 0.06) |
| LVPWs (mm) | 0.00 (-0.10, 0.10) | -0.01 (-0.11, 0.09) | -0.01 (-0.11, 0.08) | 0.05 (-0.10, 0.20) | 0.03 (-0.12, 0.18) | 0.08 (-0.07, 0.22) | 0.06 (-0.12, 0.23) | 0.02 (-0.16, 0.20) | 0.03 (-0.15, 0.21) |
| RWT (%) | **-0.56 (-1.09, -0.03)** | **-0.64 (-1.19, -0.09)** | **-0.55 (-1.07, -0.03)** | 0.19 (-0.63, 1.01) | 0.12 (-0.70, 0.94) | 0.38 (-0.41, 1.16) | -0.29 (-1.27, 0.68) | -0.52 (-1.52, 0.48) | -0.22 (-1.18, 0.73) |
| EDV (ml) | **3.03 (1.54, 4.52) ^#^** | **2.98 (1.50, 4.46) ^#^** | **2.63 (1.18, 4.07) ^#^** | 0.25 (-2.04, 2.54) | -0.18 (-2.39, 2.04) | -0.08 (-2.26, 2.09) | **3.07 (0.34, 5.80) ^#^** | **2.83 (0.13, 5.52)** | 1.95 (-0.69, 4.60) |
| ESV (ml) | **1.20 (0.58, 1.83) ^#^** | **1.21 (0.58, 1.84) ^#^** | **1.02 (0.39, 1.64) ^#^** | 0.01 (-0.96, 0.97) | -0.11 (-1.06, 0.84) | 0.10 (-0.84, 1.03) | **1.40 (0.25, 2.55) ^#^** | **1.42 (0.26, 2.58) ^#^** | 0.86 (-0.28, 2.00) |
| LVMI (g/cm^2.7^) | 0.50 (-0.29, 1.30) | 0.47 (-0.34, 1.28) | 0.52 (-0.26, 1.31) | -0.13 (-1.35, 1.09) | -0.33 (-1.55, 0.88) | 0.03 (-1.16, 1.21) | 0.65 (-0.81, 2.10) | 0.45 (-1.03, 1.93) | 0.49 (-0.95, 1.93) |
| **LV Function** |  |  |  |  |  |  |  |  |  |
| SV (ml) | 0.32 (-1.79, 2.42) | 0.18 (-1.94, 2.30) | -0.38 (-2.50, 1.75) | 1.36 (-1.87, 4.59) | 1.24 (-1.93, 4.41) | 1.97 (-1.20, 5.14) | **4.31 (0.40, 8.23) ^#^** | **4.23 (0.32, 8.15)** | **4.82 (0.87, 8.77)** |
| CO (L/min) | -0.03 (-0.22, 0.17) | -0.05 (-0.25, 0.15) | -0.09 (-0.29, 0.11) | 0.21 (-0.09, 0.51) | 0.20 (-0.09, 0.50) | 0.28 (-0.02, 0.58) | 0.32 (-0.05, 0.69) | 0.32 (-0.04, 0.69) | 0.36 (-0.02, 0.73) |
| LVEF (%) | -0.09 (-0.76, 0.58) | -0.14 (-0.84, 0.55) | -0.01 (-0.72, 0.69) | 0.04 (-0.99, 1.07) | 0.03 (-1.01, 1.06) | -0.26 (-1.32, 0.81) | -0.26 (-1.49, 0.97) | -0.39 (-1.65, 0.87) | -0.33 (-1.62, 0.96) |
| LVFS (%) | 0.00 (-0.53, 0.53) | -0.04 (-0.58, 0.50) | 0.06 (-0.49, 0.62) | 0.01 (-0.80, 0.82) | -0.01 (-0.82, 0.81) | -0.24 (-1.07, 0.59) | -0.19 (-1.16, 0.77) | -0.29 (-1.28, 0.70) | -0.26 (-1.28, 0.75) |
| E/A | 0.02 (-0.04, 0.07) | 0.02 (-0.03, 0.08) | 0.01 (-0.04, 0.07) | -0.07 (-0.15, 0.02) | -0.07 (-0.16, 0.01) | -0.08 (-0.17, 0.01) | 0.02 (-0.09, 0.12) | 0.01 (-0.10, 0.11) | 0.00 (-0.10, 0.11) |
| Tei index (%) | 1.13 (0.08, 2.18) | 0.87 (-0.22, 1.95) | 0.97 (-0.15, 2.10) | 0.28 (-1.37, 1.92) | 0.24 (-1.41, 1.89) | 0.28 (-1.44, 2.00) | -0.63 (-2.59, 1.34) | -1.01 (-3.02, 0.99) | -0.48 (-2.57, 1.61) |

The beta values and 95% CI derived from the multiple linear regression models are presented in the table. Bold: P < 0.05; #: FDR < 0.05.

Model 1: Adjusted for maternal age, income, educational level, pre-pregnancy body mass index, gender of children and postnatal SHS exposure

Model 2: Model 2 additionaly adjusted for birthweight and BMI at 4 years old.

LV Left ventricle, LVIDd LV internal diameter in diastole, LVIDs LV internal diameter in systole, IVSs Interventricular septal thickness in systole, IVSd Interventricular septal thickness in diastole, LVPWs LV posterior wall thickness in systole, LVPWd LV posterior wall thickness in diastole, EDV End diastolic volume, ESV End systolic volume, IVSd Interventricular septal thickness in diastole, IVSs Interventricular septal thickness in systole, RWT Relative wall thickness, LVMI LV mass index, SV Stroke volume, CO Cardiac output, LVEF LV Ejection fraction, LVFS LV fractional shortening.

^a^Reference category are mothers with no SHS exposure during pregnancy.

Table S3. Sensitivity analysis: Exclude mothers with GDM or HDP (N = 893)

|  | Maternal passive smoking during pregnancy^a^ | | | Maternal passive smoking during early pregnancy^b^ | | | Maternal passive smoking during mid and late pregnancy^b^ | | |
| --- | --- | --- | --- | --- | --- | --- | --- | --- | --- |
|  | crude model | model 1 | model 2 | crude model | model 1 | model 2 | crude model | model 1 | model 2 |
| **LV structure** |  |  |  |  |  |  |  |  |  |
| LVIDd (mm) | **0.43 (0.07, 0.78)** | **0.42 (0.07, 0.77)** | **0.41 (0.07, 0.75)** | **0.44 (0.08, 0.79)** | **0.47 (0.12, 0.82)** | **0.43 (0.09, 0.78)** | -0.18 (-0.58, 0.21) | -0.20 (-0.59, 0.18) | -0.18 (-0.56, 0.21) |
| LVIDs (mm) | 0.26 (-0.02, 0.53) | 0.27 (-0.02, 0.55) | 0.26 (-0.01, 0.54) | **0.29 (0.01, 0.57)** | **0.33 (0.05, 0.61)** | 0.27 (-0.01, 0.55) | -0.15 (-0.46, 0.15) | -0.15 (-0.46, 0.16) | -0.10 (-0.41, 0.21) |
| IVSd (mm) | 0.02 (-0.05, 0.09) | -0.01 (-0.09, 0.06) | 0.01 (-0.06, 0.08) | 0.03 (-0.04, 0.10) | 0.00 (-0.08, 0.08) | 0.02 (-0.06, 0.09) | 0.02 (-0.06, 0.10) | -0.02 (-0.10, 0.07) | 0.01 (-0.07, 0.09) |
| IVSs (mm) | 0.06 (-0.08, 0.20) | 0.02 (-0.12, 0.16) | 0.02 (-0.12, 0.17) | 0.09 (-0.05, 0.23) | 0.05 (-0.09, 0.19) | 0.04 (-0.10, 0.19) | 0.01 (-0.14, 0.17) | -0.04 (-0.20, 0.12) | -0.03 (-0.19, 0.13) |
| LVPWd (mm) | 0.02 (-0.11, 0.16) | -0.03 (-0.17, 0.11) | -0.02 (-0.16, 0.12) | 0.06 (-0.08, 0.19) | 0.01 (-0.13, 0.16) | 0.02 (-0.12, 0.16) | -0.02 (-0.18, 0.13) | -0.09 (-0.25, 0.07) | -0.05 (-0.20, 0.11) |
| LVPWs (mm) | 0.01 (-0.07, 0.09) | -0.03 (-0.11, 0.06) | -0.01 (-0.09, 0.08) | 0.00 (-0.08, 0.09) | -0.03 (-0.12, 0.06) | -0.02 (-0.11, 0.06) | 0.01 (-0.08, 0.10) | -0.03 (-0.13, 0.06) | 0.01 (-0.09, 0.10) |
| RWT (%) | -0.20 (-0.65, 0.25) | -0.41 (-0.88, 0.05) | -0.26 (-0.71, 0.18) | -0.19 (-0.65, 0.26) | -0.41 (-0.89, 0.06) | -0.30 (-0.75, 0.15) | 0.19 (-0.32, 0.69) | -0.05 (-0.57, 0.47) | 0.17 (-0.33, 0.67) |
| EDV (ml) | **1.36 (0.08, 2.64)** | 1.27 (-0.01, 2.55) | 1.23 (-0.03, 2.49) | **1.38 (0.08, 2.67)** | **1.43 (0.13, 2.73)** | **1.28 (0.00, 2.56)** | -0.66 (-2.09, 0.77) | -0.79 (-2.20, 0.63) | -0.67 (-2.09, 0.74) |
| ESV (ml) | 0.50 (-0.04, 1.03) | 0.51 (-0.04, 1.06) | 0.51 (-0.03, 1.05) | **0.57 (0.03, 1.11)** | **0.64 (0.08, 1.19)** | 0.52 (-0.03, 1.07) | -0.35 (-0.94, 0.25) | -0.35 (-0.95, 0.26) | -0.23 (-0.84, 0.37) |
| LVMI (g/cm^2.7^) | 0.23 (-0.45, 0.92) | 0.07 (-0.64, 0.79) | 0.31 (-0.38, 1.01) | 0.35 (-0.35, 1.04) | 0.25 (-0.48, 0.97) | 0.39 (-0.31, 1.10) | -0.07 (-0.83, 0.70) | -0.29 (-1.08, 0.49) | 0.08 (-0.69, 0.86) |
| **LV Function** |  |  |  |  |  |  |  |  |  |
| SV (ml) | 0.02 (-1.79, 1.82) | -0.22 (-2.06, 1.62) | 0.03 (-1.83, 1.89) | 0.05 (-1.78, 1.87) | -0.14 (-2.00, 1.72) | -0.17 (-2.05, 1.72) | -0.86 (-2.86, 1.14) | -0.87 (-2.89, 1.15) | 0.08 (-1.98, 2.15) |
| CO (L/min) | -0.03 (-0.20, 0.14) | -0.05 (-0.22, 0.12) | -0.02 (-0.20, 0.15) | -0.04 (-0.21, 0.13) | -0.05 (-0.23, 0.12) | -0.06 (-0.23, 0.12) | -0.05 (-0.24, 0.13) | -0.03 (-0.22, 0.15) | 0.04 (-0.15, 0.24) |
| LVEF (%) | 0.07 (-0.49, 0.63) | 0.00 (-0.60, 0.59) | 0.00 (-0.60, 0.61) | -0.03 (-0.60, 0.54) | -0.12 (-0.72, 0.48) | 0.02 (-0.60, 0.63) | 0.13 (-0.50, 0.76) | 0.08 (-0.57, 0.74) | 0.00 (-0.68, 0.68) |
| LVFS (%) | 0.07 (-0.37, 0.52) | 0.02 (-0.44, 0.49) | 0.03 (-0.45, 0.50) | 0.01 (-0.43, 0.46) | -0.05 (-0.52, 0.42) | 0.06 (-0.43, 0.54) | 0.06 (-0.44, 0.55) | 0.03 (-0.48, 0.55) | -0.04 (-0.57, 0.49) |
| E/A | 0.02 (-0.03, 0.06) | 0.01 (-0.04, 0.06) | 0.00 (-0.05, 0.05) | 0.04 (-0.01, 0.09) | 0.04 (-0.01, 0.09) | 0.03 (-0.03, 0.08) | -0.01 (-0.06, 0.05) | -0.02 (-0.07, 0.04) | -0.03 (-0.08, 0.03) |
| Tei index (%) | 0.69 (-0.21, 1.59) | 0.40 (-0.53, 1.34) | 0.52 (-0.46, 1.50) | 0.66 (-0.25, 1.57) | 0.33 (-0.62, 1.28) | 0.44 (-0.55, 1.44) | -0.05 (-1.06, 0.95) | -0.27 (-1.30, 0.77) | -0.15 (-1.24, 0.94) |

The beta values and 95% CI derived from the multiple linear regression models are presented in the table. Bold: P < 0.05; #: FDR < 0.05.

Model 1: Adjusted for maternal age, income, educational level, pre-pregnancy body mass index, gender of children and postnatal SHS exposure

Model 2: Model 2 additionaly adjusted for birthweight and BMI at 4 years old.

LV Left ventricle, LVIDd LV internal diameter in diastole, LVIDs LV internal diameter in systole, IVSs Interventricular septal thickness in systole, IVSd Interventricular septal thickness in diastole, LVPWs LV posterior wall thickness in systole, LVPWd LV posterior wall thickness in diastole, EDV End diastolic volume, ESV End systolic volume, IVSd Interventricular septal thickness in diastole, IVSs Interventricular septal thickness in systole, RWT Relative wall thickness, LVMI LV mass index, SV Stroke volume, CO Cardiac output, LVEF LV Ejection fraction, LVFS LV fractional shortening.

^a^Reference category are mothers with no SHS exposure during pregnancy.

^b^Reference category are mothers with no SHS exposure at each period.

Table S4. Sensitivity analysis: Replacing BMI with Body Surface Area (BSA) in Model 2 (N = 1089)

|  | Passive smoking during pregnancy^a^ | Passive smoking during early pregnancy^b^ | Passive smoking during mid or late pregnancy^b^ |
| --- | --- | --- | --- |
| **LV structure** |  |  |  |
| LVIDd (mm) | **0.32 (0.03, 0.62)** | **0.40 (0.11, 0.70)** | -0.17 (-0.49, 0.15) |
| LVIDs (mm) | 0.22 (-0.02, 0.46) | **0.26 (0.01, 0.50)** | -0.06 (-0.33, 0.20) |
| IVSd (mm) | 0.01 (-0.05, 0.08) | 0.00 (-0.07, 0.07) | 0.03 (-0.04, 0.10) |
| IVSs (mm) | 0.01 (-0.11, 0.14) | 0.02 (-0.10, 0.15) | -0.04 (-0.18, 0.10) |
| LVPWd (mm) | -0.02 (-0.14, 0.11) | 0.02 (-0.11, 0.14) | -0.01 (-0.15, 0.12) |
| LVPWs (mm) | 0.00 (-0.07, 0.07) | -0.02 (-0.10, 0.05) | 0.03 (-0.05, 0.11) |
| RWT (%) | -0.17 (-0.58, 0.24) | -0.33 (-0.75, 0.08) | 0.28 (-0.17, 0.73) |
| EDV (ml) | 0.96 (-0.11, 2.03) | **1.23 (0.15, 2.31)** | -0.67 (-1.85, 0.51) |
| ESV (ml) | 0.43 (-0.03, 0.90) | **0.51 (0.04, 0.98)** | -0.16 (-0.68, 0.35) |
| LVMI (g/cm^2.7^) | 0.38 (-0.24, 1.00) | 0.49 (-0.14, 1.12) | -0.22 (-0.91, 0.47) |
| **LV Function** |  |  |  |
| SV (ml) | 0.03 (-1.62, 1.68) | -0.51 (-2.18, 1.16) | 0.62 (-1.19, 2.43) |
| CO (L/min) | 0.01 (-0.15, 0.17) | -0.07 (-0.23, 0.09) | 0.12 (-0.05, 0.30) |
| LVEF (%) | -0.09 (-0.64, 0.46) | -0.04 (-0.60, 0.52) | -0.18 (-0.79, 0.43) |
| LVFS (%) | -0.06 (-0.50, 0.37) | -0.01 (-0.45, 0.43) | -0.19 (-0.67, 0.28) |
| E/A | -0.01 (-0.05, 0.04) | 0.01 (-0.03, 0.06) | -0.02 (-0.07, 0.02) |
| Tei index (%) | 0.53 (-0.35, 1.42) | 0.55 (-0.35, 1.44) | -0.08 (-1.05, 0.89) |

The beta values and 95% CI derived from the multiple linear regression models are presented in the table. Bold: P < 0.05; #: FDR < 0.05.

Model 2: Adjusted for maternal age, income, educational level, pre-pregnancy body mass index, gender of children, postnatal SHS exposure, birthweight and BMI at 4 years old.

LV Left ventricle, LVIDd LV internal diameter in diastole, LVIDs LV internal diameter in systole, EDV End diastolic volume, ESV End systolic volume, IVSd Interventricular septal thickness in diastole, IVSs Interventricular septal thickness in systole, RWT Relative wall thickness, LVMI LV mass index, SV Stroke volume, CO Cardiac output, LVEF LV Ejection fraction, LVFS LV fractional shortening.

^a^Reference category are mothers with no SHS exposure during pregnancy.

^b^Reference category are mothers with no SHS exposure at each period.
